# Hyaluronic Acid-Functionalized Liposomes for Co-delivery of 5-Fluorouracil and Cannabidiol Against Colorectal Cancer

**DOI:** 10.34172/apb.025.43401

**Published:** 2025-10-15

**Authors:** Soheil Abbaspour-Ravasjani, Mahdi Zeinali, Leila Asadollahi, Malahat Safavi, Amin Mahoutforoush, Mehdi Talebi, Hamed Hamishehkar

**Affiliations:** ^1^Student Research Committee, Department of Medical Nanotechnology, School of Medicine, Zanjan University of Medical Sciences, Zanjan, Iran; ^2^Drug Applied Research Center, Tabriz University of Medical Sciences, Tabriz, Iran; ^3^Department of Food Industry Science and Engineering, Faculty of Agriculture, University of Tabriz, Tabriz, Iran; ^4^Department of Applied Cell Sciences, Faculty of Advanced Medical Sciences, Tabriz University of Medical Sciences, Tabriz, Iran; ^5^New Material and Green Chemistry Research Center, Khazar University, 41 Mehseti Street, Baku, AZ1096, Azerbaijan

**Keywords:** 5-fluorouracil, Colorectal cancer, Cannabidiol, Potential targeted drug delivery system, Liposomes

## Abstract

**Purpose:**

Colorectal cancer (CRC) is a formidable global health challenge, ranking as the third most prevalent cancer. Conventional treatments like surgery, radiation, and chemotherapy are limited by adverse effects, driving the search for more effective alternatives.

**Methods:**

This study investigates the synergistic potential of co-delivering 5-fluorouracil (5-FU) and cannabidiol (CBD) using hyaluronic acid (HA)-decorated liposomes. While 5-FU is a cornerstone of CRC treatment, CBD offers promise as an anti-tumor agent. The HA-decorated liposomes enable potential targeted drug delivery to CD44 receptors, which are overexpressed in CRC, while minimizing systemic toxicity by reducing the concentrations of anticancer drugs required.

**Results:**

The liposomal formulation displays optimal physicochemical properties (a sub100nm size and an appropriate negative zeta potential) and acceptable encapsulation and loading efficiencies, ensuring effective drug release. In vitro studies demonstrate that the targeted liposomes have superior anticancer effects, inducing apoptosis (up to 59.1%), cell cycle arrest in the Sub-G1 and G0-G1 phases, reduction of cell viability to 6.98% in human colorectal adenocarcinoma (HT-29) cells, induction of oxidative stress, and inhibition of colony formation. Additionally, HepG2 (non-CD44-expressing) cells were used as a control to evaluate CD44-targeting efficiency. Gene expression analysis by real-time PCR indicates modulation of key genes associated with cell cycle progression and apoptosis.

**Conclusion:**

This multifaceted approach presents a promising strategy for CRC therapy, but requires additional optimization and rigorous in vivo investigations to facilitate successful clinical translation. In particular, optimization of drug-release kinetics and thorough in vivo validation are essential to advance this platform toward clinical application.

## Introduction

 Mortality from cancer remains a major global health challenge, and colorectal cancer (CRC) ranks among the most prevalent malignancies and is the third leading cause of cancer-related death in both men and women.^1,2^ Additionally, CRC is the the fifth most common cancer in men and the third in women.^3^ However, current standard treatments for CRC are surgery, radiotherapy, and chemotherapy, which are limited by significant adverse effects, underscoring the need for more effective therapeutic strategies. Selection of the optimal treatment depends on disease stage, patient performance status, and tumor molecular profile.^4^ As such, approaches that increase efficacy while minimizing toxicity are urgently required. Before the mid-1990s, 5-fluorouracil (5-FU), despite its constrained efficacy, was the sole drug available for metastatic CRC. The subsequent introduction of oxaliplatin and irinotecan, combined with 5-FU, markedly improved outcomes for metastatic CRC.^5^ Additionally, 5-FU has long been used to treat CRC for many years. Cannabidiol (CBD), a phytocannabinoid first isolated by Roger Adams in 1940, is one of the 113 identified cannabinoids in hemp.^6^ Recent studies demonstrate that CBD inhibits CRC growth while sparing healthy cells from apoptosis.^7^ Its anti-tumor mechanisms include blocking pro-survival and proliferative signaling pathways, reducing dependence on CB1/CB2 receptors, inducing apoptosis, generating reactive oxygen species (ROS), and arresting the cell cycle at multiple stages.^8^ Nanoparticle-based delivery systems for 5-FU have explored both passive and active targeting strategies.^9,10^ Among these, liposomes stand out as versatile carriers for therapeutic and imaging agents.^11^ Cationic lipids and polymers are widely used as non-viral vectors due to their scalability, high drug-loading capacity, and suitability for co-delivery in cancer therapy. However, their toxicity remains a limitation, prompting the development of safer cationic compounds.^12^ Chitosan (CS), a natural amino polysaccharide, facilitates cellular transport, and has shown promise in gene transfection.^13^ For example, Liu and Yao reported that CS and trimethylated CS oligomers were non-toxic to cells, unlike the cationic lipid DOTAP, which reduced cell viability by 50%.^14,15^ Hyaluronic acid (HA), a targeting ligand for CRC, binds to CD44 receptors overexpressed in cancer cells (e.g., HT-29 colorectal adenocarcinoma).^16^ HA-conjugated liposomes enhance drug delivery by improving target specificity via CD44-mediated internalization, prolonging circulation time, and optimizing biodistribution. The electrostatic bonding method for HA-liposome conjugation offers a simple, controllable alternative to covalent bonding.^17^

 Recent evidence supports the hypothesis that nanoparticle base co-delivery of 5-FU and CBD could enhance 5-FU bioavailability, mitigate drug resistance, and amplify anti-tumor efficacy.^18^ However, a non-toxic, targeted delivery system is critical for simultaneous administration. In this study, we developed HA-decorated liposomes to co-deliver 5-FU (a conventional chemotherapeutic) and CBD (a natural anti-tumor adjuvant) for CRC treatment. Our goals were to (a) evaluate synergistic anti-CRC effects *in vitro*, and (b) minimize off-target toxicity associated with cationic liposomes.

## Material and Methods

###  Materials

 The 5-FU powder was procured from Cedal Pharmaceutical Group (Tehran, IRI), and CBD was obtained from Cayman Chemical Co. Ltd (Ann Arbor, MI). Additionally, the following materials were sourced: Phosphate-buffered saline tablets (PBS), 3-(4,5-dimethylthiazol-2-yl)-2,5-diphenyltetrazolium bromide (MTT), RPMI-1640 medium (R5886), dimethyl sulfoxide (DMSO, 99.9%), cholesterol (from lanolin, ≥ 99.0%), glycerol monostearate (GMS), 4’,6-diamidino-2-phenylindole (DAPI), trypsin (0.25% EDTA solution), fetal bovine serum (FBS), CS (medium molecular weight), 1,2-diacyl-sn-glycero-3-phosphocholine (lecithin, 94%), 2′,7′-dichlorofluorescin diacetate (DCFH-DA, ≥ 97%), acetonitrile (ACN), Tween^®^ 80, fluorescein sodium salt, and HA (low viscosity, low endotoxin) were all purchased from Sigma Aldrich Co. (Hamburg, Germany). Additionally, the Annexin V-FITC/PI flow cytometry kit was procured from Exbio Co. (Prague, Czech Republic).

###  Preparation of liposomes 

 The liposomal formulations were prepared using the thin-film hydration method as previously described ^19^. Initially, nanoliposomes were prepared to evaluate the feasibility of using HA as a CRC targeting ligand. Both non-decorated and HA-decorated liposomes were formulated from CS, GMS, cholesterol, and lecithin. In this study, GMS was used to establish a negative charge on the liposome’s surface, thereby giving chitosan coating ability to the liposome. Before coating the liposomes, the amount of GMS in the liposome composition was optimized. The optimal GMS amount was assessed (0, 20, 40, and 80 mg) by evaluating the stability of the resulting suspensions; particle size, polydispersity index (PDI), and zeta potential were used as stability indicators. Based on these measurements, 40 mg GMS was selected as the optimal amount. Liposomal formulations were prepared in three phases. In the first phase, different amounts of GMS were incorporated to obtain a negative surface charge. As shown in Table 1, the quantities of lecithin, cholesterol, and Tween^®^ 80 were held constant while the amount of GMS varied between formulations.

**Table 1:** Components of first phase formulations

**Formulation**	**Lecithin (mg)**	**Cholesterol (mg)**	**Tween 80 (mg)**	**GMS (mg)**
F1	90	10	20	0
F2	90	10	20	20
F3	90	10	20	40
F4	90	10	20	80

All other ingredients were constant except GMS due to reaching an appropriate negative surface charge on liposomes.

###  Decorating prepared liposomes with HA

 After preparing each lipid composition according to Table 1, the lipids were dissolved in 20 mL of chloroform. The lipid solution was transferred into a round-bottom flask, and the solvent was evaporated under vacuum at 45°C using a rotary evaporator (Heidolph, Hei-VAP Core, Germany) to produce a thin lipid film. The film was subsequently hydrated with 10 ml of PBS (pH = 7.4). The resulting liposomal suspension was homogenized (Heidolph, SilentCrusher, Germany) at 65 °C for 20 minutes at 15,000 rpm to reduce particle size.

 After 80mg GMS was selected as the optimal amount for the nanoliposomes, the second phase, coating the liposomes with CS to confer a positive charge and enable HA adsorption, was performed. Based on our previous publication, CS was used to coat the liposomes.^20^ To prepare the coating solution, 100 mg of CS was dissolved in 10 mL of 1% acetic acid; 0.5 mL of this CS solution was then added dropwise to 10 mL of liposomal suspension and mixed for 1 hour. Finally, to surface decoration of the liposomes with HA, 0.195 mL of a 100 mg/mL HA solution was added dropwise to the CS-coated liposomal suspension.

###  Drug loading 

 In the final phase, to achieve the best anticancer activity, various ratios of 5-FU and CBD were encapsulated in the liposomes (Table 2). CBD was incorporated by adding the required amounts to the lipid solution prior to thin-film formation, whereas 5-FU was dissolved in PBS and used to hydrate the lipid film during thin-film hydration.

**Table 2:** Represents the ratio of drugs in each formulation

**Formulation**	**Lecithin (mg)**	**Cholesterol (mg)**	**Tween 80 (mg)**	**GMS (mg)**	**5-FU (mg)**	**CBD (mg)**
FD1	90	10	20	80	10	0
FD2	90	10	20	80	0	10
FD3	90	10	20	80	9.75	0.314
FD4	90	10	20	80	9.75	0.628
FD5	90	10	20	80	9.75	1.257
FD6	90	10	20	80	9.75	2.515
FD7	90	10	20	80	9.75	5.031

###  Optimization and characterization of liposomes

 To optimize the GMS amount (0mg, 20mg, 40mg, 80mg) and compare formulation characteristics, samples were diluted 1:10 with filtered deionized water prior to measurement. particle size, PDI, and zeta potential were measured by dynamic light scattering (DLS) using a Zetasizer Nano ZS (Malvern, UK) for formulations containing different GMS amounts, for CS-coated liposomes, and for liposomes coated with both CS and HA.

 Nanoparticle morphology was examined by Tescan VEGA II XMU scanning electron microscope (SEM). Chemical characterization and possible interactions among formulation components were analyzed by Fourier transform spectroscopy (FTIR). For FTIR analysis, samples (as described in Table 3) were prepared as KBr pellets and spectra were recorded using a TENSOR 27, Brucker FT-IR spectrometer. spectra were collected over the range of 4000–400 cm^-1^.

**Table 3:** Components of formulations

**Contents**	**F1**	**F2**	**F3**	**F4**	**F5**	**F6**
cholesterol	10 mg	10 mg	10 mg	10 mg	10 mg	10 mg
lecithin	90 mg	90 mg	90 mg	90 mg	90 mg	90 mg
Tween 80	20 mg	20 mg	20 mg	20 mg	20 mg	20 mg
GMS	-	80 mg	80 mg	80 mg	80 mg	80 mg
CBD	-	-	1.25 mg	1.25 mg	1.25 mg	1.25 mg
5-FU	-	-	-	9.75 mg	9.75 mg	9.75 mg
CS	-	-	-	-	5 mg	5 mg
HA	-	-	-	-	-	19.5 mg

###  Encapsulation measurement 

 High-performance liquid chromatography (HPLC) was used to determine the encapsulation efficiency (EE) of 5-FU and CBD in the liposomes. To separate free (unencapsulated) drug from encapsulated drug, 2 mL of each liposomal formulation was diluted with ethanol to yield a final ethanol concentration of 40% (v/v), ensuring complete dissolution of unencapsulated 5-FU and CBD. The diluted sample was then filtered using an Amicon^®^ Ultra-Centrifugal Filter unit (30 KD MWCO; Merck Millipore, Germany) and centrifuged at 4000 rpm for 10 min. The filtrate accumulated in the lower chamber of the unit was collected for analysis.

 The mobile phase was prepared as acetonitrile:water (ACN:water, 75:25 v/v), degassed through a 0.45 μm filter and sonicated for 30 minutes prior use. Sample components were separated on C18 column (5 μm, 4.6 × 250 mm; Knauer, Berlin, Germany). The mobile phase flow rate was set to 1.5 mL/min and the detection was performed with a UV detector at 214 and 265 nm. For quantification, 40 μL of the 1:10 diluted sample (collected from the bottom of the filter unit) was injected into the HPLC. EE and Loading efficiency (LE) were calculated using below equations.


$$EE=\frac{Initially\;total\;drug\;taken-Unloaded\;drug}{Initially\;total\;drug\;taken}\times 100$$

$$LE=\frac{Total\;amount\;of\;entrapped\;drug}{Total\;amount\;of\;liposomes}\times 100$$

###  In vitro release of 5-FU and CBD

 The dialysis-bag diffusion method was used to determine *in vitro* release profiles of 5-FU and CBD from co-loaded liposomes.^21^ Two milliliters of the liposomal suspension were mixed with 2 ml PBS and transferred into a dialysis bag (MWCO 20 kDa). The dialysis bag was immersed in 25 mL of PBS (10 mM) at pH 7.4 and 5.5 containing 1% (v/v) Tween^®^ 80 to maintain sink conditions. The release system was maintained at 37 °C under agitation at 300 RPM. At predetermined time points, 1 mL aliquots of the external medium were withdrawn and immediately replaced with 1 mL fresh buffer. Released 5-FU and CBD concentrations were quantified by HPLC as described above.

###  Drug release kinetics

 To elucidate the drug release mechanisms, the experimental release data were fitted to various kinetic models including zero-order, first-order, second-degree polynomial, Higuchi, Korsmeyer-Peppas, and diffusion-relaxation models.^22^ Since drug release models are primarily semi-empirical, this evaluation was performed using MathCAD 15.0 software, with the best-fit model selected based on the values of R2 and n. Table 4 presents these mathematical and semi-empirical models commonly employed to analyze drug release mechanisms. Each model provides distinct insights: zero-order kinetics reflects a constant release rate independent of drug concentration, where Qt is the cumulative drug released at time t, Q0 is the initial amount, and K0 is the rate constant; first-order model describing concentration-dependent release; second-degree polynomial model a quadratic fit for empirical non-linear profiles; Higuchi model representing diffusion-controlled release from matrix systems, with KH as the Higuchi constant and C as intercept; Korsmeyer-Peppas model, where Q∞ is release at infinity, KkP is the kinetic constant, and n indicates the release mechanism (Values of n = 0.43, 0.43 < n < 0.85, n = 0.85, and n > 0.85 correspond to Fickian diffusion, non-Fickian (anomalous) transport, Case II (relaxational) transport, and Super Case II transport mechanisms, respectively); and the diffusion-relaxation model and the diffusion-relaxation model combines the effects of diffusion and matrix relaxation or swelling, accounting for both Fickian diffusion (Kd term) and relaxation/erosion (Kr term).

**Table 4:** Kinetics models

**Model name**	**Equation**
Zero order	$Q_{t}=Q_{0}+K_{0}t$
First order	$\ln Q_{t}=\ln Q_{0}+K_{1}t$
Second degree polynomial	$Q_{t}=Q_{0}+K_{s1}t+K_{s2}t^{2}$
Higuchi	$Q_{t}=C+K_{H}t^{1/2}$
Korsmeyer-Peppas	$\frac{Q_{t}}{Q_{\infty }}=K_{kp}t^{n}$
Diffusion relaxation	$\frac{Q_{t}}{Q_{\infty }}=K_{d}t^{n}+K_{t}t^{2n}$

###  Formulation stability

 The stability of targeted mix-liposomes was evaluated by monitoring changes in particle size, PDI, and drug leakage over time. Formulations were stored in sealed vials under refrigerated conditions (4–8 °C) for a period of 60 days. At predetermined intervals (0, 15, 30, 45 and 60 days), samples were withdrawn and analyzed for particle size and PDI using dynamic light scattering, while drug retention was assessed by measuring the percentage of entrapped drug relative to the initial loading.

###  Hemolysis assay

 To assess the *in vivo* safety of HA-decorated liposomes, a hemolysis assay was performed ^23^. Hemolysis defined as release of intracellular hemoglobin from red blood cells (RBCs) was quantified by measuring the percentage of hemoglobin released after incubation with nanoliposomes. Human whole blood was collected into a sterile tube and centrifuged at 1500 rpm for 5 minutes. The plasma and buffy coat were removed and the RBC pellet was washed and resuspended in PBS. Liposomal suspensions were diluted with PBS to obtain final concentrations of 0, 2, 4, 8, 16, 32, 64 mg/mL in a total volume of 1 ml. RBCs in PBS served as the negative control, and 1% Triton X-100 mixed with RBCs served as the positive control. Samples after mixing with RBCs were incubated at 37°C for 1 h, then centrifuged at 1500 rpm for 5 min. the absorbance of the supernatant was measured at 450 nm and the percentage of hemolysis was calculated using the below equation


$$Hemolysis\;(\%)=\frac{Absorbance\left(sample\right)-Absorbance\left(PBS\right)}{Absorbance\left(1\%Triton\;X-100\right)-Absorbance\left(PBS\right)}\times 100$$

###  Cell culture

 HT-29 cells were obtained from Pasteur Institute (Tehran, IRI), and cultured with RPMI supplemented with 10% FBS. Cells were maintained at 37 °C in a humidified atmosphere containing 5% CO_2_. Cells were sub-cultured 24 and 48 h after seeding at an initial concentration 4 × 10^4^ cells/mL. Experiments (MTT assay, flow cytometry, and fluorescence imaging) were performed when cultures reached ~70% confluence.

###  Cellular uptake

 Cellular uptake of targeted nanoparticles by HT-29 and HEP-G2 cells was assessed by flow cytometry (BD FACSCalibur, Franklin Lakes, NJ). Cells were seeded in 12-well plates at 4 × 10^4^ cells/well and incubated overnight. Fluorescent-labeled liposome formulations were applied at their respective IC_50_ concentrations.

 At 1, 3, 6, 12, and 24 h after treatment, culture medium was removed, cells were trypsinized, washed twice with PBS, and analyzed by flow cytometry. Mean fluorescent intensity was recorded and data were analyzed using the FlowJo software.

###  Cell viability assay

 The MTT assay was used to evaluate the effects of 5-FU and CBD on HT-29 cell proliferation and viability. Cells were seeded in a 96-well plates at 1.2 × 10^4^ cells/well and incubated overnight. To determine the IC_50_ values, cells were exposed to serial dilution of free drugs and liposomal formulations of the drugs for 48 h. Following the determination of IC50 values for liposomal CBD and liposomal 5-FU, the most effective combination ratio of CBD and 5-FU was identified by maintaining the IC50 concentration of liposomal 5-FU as a fixed value. Subsequently, freshly seeded cells were exposed for 48 hours to varying molar ratios of 5-FU to CBD (75:0, 75:1, 75:2, 75:4, 75:8, and 75:16 µM:µM). After each treatments finishing the incubation time, treatment medium was replaced with 100 μL fresh medium containing 50 μL MTT solution (2 mg/mL in PBS) and plates were incubated for 4 h at 37 °C. Formazan crystals were solubilized with 100 μL DMSO and absorbance was measured at 570 nm using a microplate reader (Sunrise^TM^, Tecan, Switzerland)

###  Cellular ROS detection

 Intercellular ROS generation was measured using the DCFH-DA and a flow cytometry. HT-29 cells were seeded at 4 × 10^5^ cell/well in 6-wll plates and treated with formulations at their 48-h IC_50_ concentrations. After 48 hours of treatment, cells were incubated with 10 μM DCFH-DA (solubilized in serum free RPMI) for 1 h at 37 °C, washed twice with PBS, detached, and analyzed by flow cytometry. Green fluorescence intensity was measured using the FL1-H channel.

###  Colony formation

 A colony-formation assay was performed to assess long-term anti-proliferative capacity following treatment. HT-29 cells were seeded at 2 × 10^3^ cell/well in 6-well plates and incubated for 48 h. After attaching the cells to the plates, the cells were treated with EC_10_ concentrations of the drugs and incubated for 2 weeks under standard culture conditions. Colonies were then fixed, stained with crystal violet, and photographed. The colony counts were evaluated with ImageJ software (NIH, Bethesda, MD).

###  Apoptosis assay

 Apoptosis and necrosis were evaluated using an Annexin V/propidium iodide (PI) staining kit was used. HT-29 cells were seeded at 4 × 10^5^ cell/well in 6-well plates and incubate overnight. Cells were treated with formulations at their IC_50_ concentration for 48 h, then trypsinized, washed with PBS, and re-suspended in 100 µL of binding buffer. Annexin V/PI staining was performed according to the manufacturer’s instructions. And samples were analyzed by flow cytometry.

###  DAPI staining

 DNA fragmentation was assessed by DAPI staining. HT-29 cells were seeded at 4 × 10^5^ cell/well in 6-well plates covered with 1 × 1 cm glass Lamel and incubated overnight, then they were treated with the nanoformulation at the concentration corresponding to the 48-h IC_50_ and incubated for 48 h. After removing culture medium, cells were washed with PBS and stained with a DAPI (1 μg/mL) for 15 minutes as described by Asadollahi et al.^24^ Nuclear morphology and DNA fragmentation were observed and imaged using a fluorescence microscope at 40X magnification (BX50, Olympus, Japanese).

###  Cell cycle analysis

 For cell cycle analysis, HT-29 cells at 4 × 10^5^ cell/well in 6-well plates and treated with nanoformulations or free drugs at their IC_50_ concentrations for 48 h. Cells were fixed in 70% ethanol, stained with PI according to the Zeinali et al, and analyzed by flow cytometry to determine cell cycle distribution and DNA synthesis.^25^

###  Real-time PCR assay

 Gene expression was evaluated by quantitative real-time PCR.^26^ HT-29 cells were seeded at 5 × 10^4^ and incubated overnight. Cells were treated with formulations at the 48-h IC_50_ concentration and incubated for 48 h. Total RNA was extracted using TRIzol regent (Thermo Fisher Scientific, Waltham, MA);1 mL TRIzol was added per well and incubated for a 20 min, the 200 μL chloroform was added, mixed gently, and the mixture was incubated for 20 min at -20 °C. Following centrifugation at 12,000 RPM for 15 min at 4–8 °C, the aqueous phase was collected, and RNA was precipitated with 500 μL isopropanol for 10 min. RNA pellets were washed with isopropanol, air-dried, and resuspended in 20 μL RNase-free water. RNA concentration was measured using a NanoDrop 200 spectrophotometer (Thermo Fisher Scientific).

 Complementary DNA was synthesized using the QuantiTect Reverse Transcription kit (Qiagen, Hilden, Germany). Quantitative PCR was performed using Power SYBR^®^ GREEN PCR master mix (Applied Biosystems, Foster City, CA). β-Actin was used as the internal control. Expression levels of target genes related to apoptosis and cell cycle regulation (Bax, Bcl-2, p53, Survivin, NF-κB, mTOR, TNF-α, FasL, p38, Caspase-3, Caspase-8, Caspase-9) were quantified using the 2−ΔΔCT method. Primer sequences are listed on Table 5.

**Table 5:** Primer sequences

**Gene**	**Forward**	**Reverse**	**Product size (bp)**
*β -Actin*	5′-GGTCATCACTATTGGCAACG-3′	5′-ACGGATGTCAACGTCACACT-3′	~200
*Bax*	TCCCTGGAGAAGAGCTACG	GTAGTTTCGTGGATGCCACA	~150
*Survivin*	GACCACCGCATCTCTACATTC	TGCTTTTTATGTTCCTCTATGGG	~180
*Bcl2*	5′- GGCTGGGGATGACTTCTCTC-3′	5′- ACAATCCTCCCCCAGTTCAC−3′	~220
*NF- κ B*	GAAATTCCTGATCCAGACAAAAAC	CTGGTGGACACATACAGGAAGAC	~300
*mTOR*	CTTGTTTGTGGCTCTGAATGAC	GGCACTCTGCTCTTTGATTCTT	~250
*P-53*	5′-CCTCAGCATCTTATCCGAGTGG-3′	5′-TGGATGGTGGTACAGTCAGAGC-3′	~200
*TNF- α*	CTCTTCTGCCTGCTGCACTTTG	ATGGGCTACAGGCTTGTCACTC	~180
*FasL*	5′ GCAGCCCTTCAATTACCCAT 3′	5′ CAGAGGTTGGACAGGGAAGAA 3′	~230
*P-38*	5′–AGAGTCTCTGTCGACCTGCT-3′	5′-CCTGCTTTCAAAGGACTGGT-3′	~210
*Caspase 3*	TGGTGATGAAGGGGTCATTTATG	TTCGGCTTTCCAGTCAGACTC	~190
*Caspase 8*	TGGTTCATCCAGTCGCTTTG	AATTCTGTTGCCACCTTTCG	~240
*Caspase 9*	CTGTCTACGGCACAGATGGAT	GGGACTCGTCTTCAGGGGAA	~200

###  Statistical analysis 

 All experiments were performed in biological triplicate. Statistical analyses were conducted using GraphPad Prism version 9 (GraphPad Software, San Diego, CA). Data were analyzed by an independent t-test or Two-way analysis of variance (Two-way ANOVA) where appropriate. Multiple comparisons were performed using a Tukey’s honestly significant difference (HSD) test. Differences were considered statistically significant at *P* < 0.05.

## Results

###  Characterization of Liposomes

####  Physical characterization

**Figure 1:**
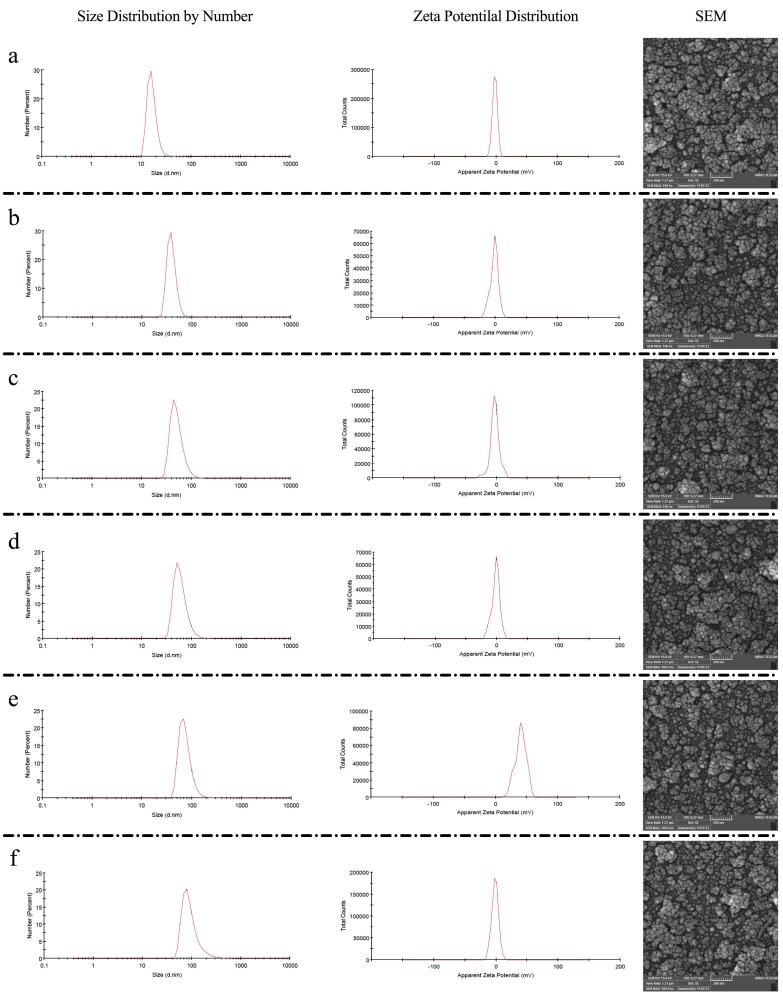
Physicochemical and morphological characterization of liposomal formulations. Left panels: particle size distribution by number for each formulation. Middle panels: zeta-potential distribution. Right panels: representative scanning electron microscopy (SEM) images. Formulations shown are: (a) no glycerol monostearate (GMS), (b) 20 mg GMS, (c) 40 mg GMS, (d) 80 mg GMS, (e) 80 mg GMS + chitosan (CS), and (f) 80 mg GMS + CS + hyaluronic acid (HA). Data illustrate a narrow sub-100 nm size distribution and progressive surface modification following CS and HA decoration. Representative SEM images are shown; scale bars are indicated in the panels

**Figure 2:**
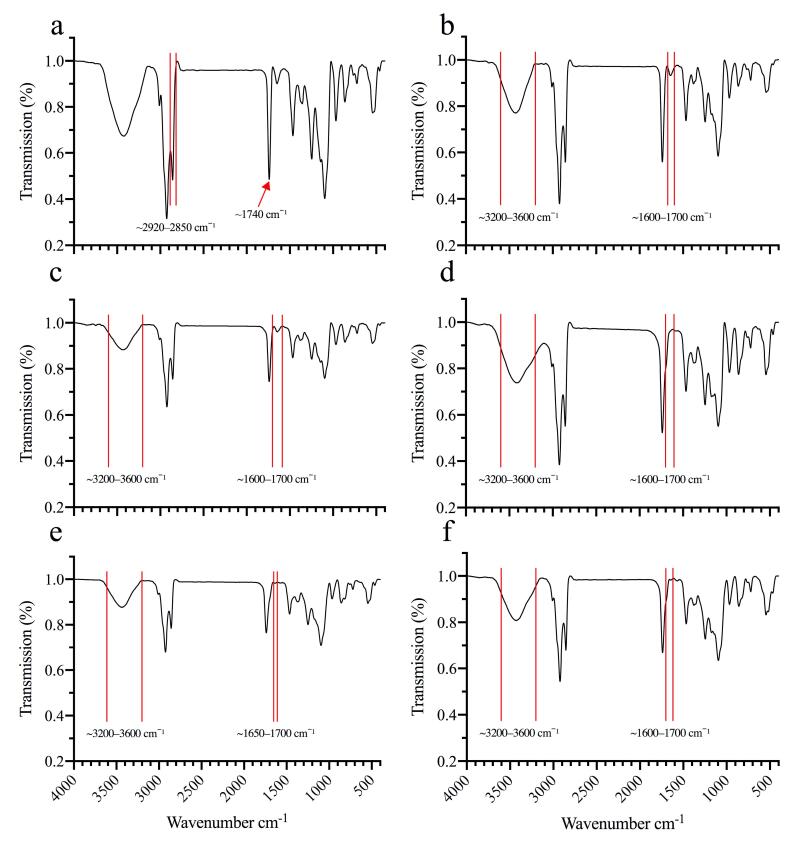
FT-IR spectra of liposomal formulations demonstrating chemical changes and interactions following component addition. Curves correspond to: (a) blank liposomes, (b) liposomes containing 80 mg glycerol monostearate (GMS), (c) GMS liposomes loaded with cannabidiol (CBD), (d) GMS liposomes loaded with CBD and 5-fluorouracil (5-FU), (e) (d) coated with chitosan (CS), and (f) (e) further decorated with hyaluronic acid (HA). Key peak shifts and band assignments that indicate drug incorporation and surface modification are annotated in the figure

 The mean diameter of blank nanoliposomes was 16.59 ± 4.70 nm. Increasing the GMS content led to a progressive increase in particle size; the optimal formulation exhibited a mean diameter of 51.57 ± 16.79 nm, with PDI values of 0.178 and 0.307, respectively. Zeta potential measurements indicated changes in surface charge with varying GMS content; the optimal formulation displayed a zeta potential of -3.38 ± 7.48 mV (Figure 1). Coating the liposomes with CS increased particle size from 60.56 nm to 76.93 nm and shifted the zeta potential from -3.38 mV to 40.10 mV. Subsequent decoration with HA increased their size to 96.35 nm and reduced the zeta potential to -1.53 mV (Figure 1). Reported for PDI values were 0.178, 0.295, 0.556, and, 0.484 for blank, GMS-loaded, CS-coated and, HA-decorated liposomes, respectively (Figures 1 and 2). SEM images showed spherical, uniform shaped nanoparticles (Figures 1 and 2).

 EE and LE were determined for each formulation. For single-drug formulations, EE was 95.1 ± 6.16% for 5-FU and 98.2 ± 2.43%, for CBD. In mixed-drug liposomes (5-FU:CBD molar ratio 75:4), EE was 94.1 ± 3.26% for 5-FU and 97.2 ± 4.21% for CBD. LE values were 4.75 ± 0.30% for 5-FU and 4.91 ± 0.12% for CBD in single-drug systems; in mixed liposomes, LE was 4.58 ± 0.15% for 5-FU and 0.61 ± 0.005% for CBD.

####  Fourier Transform Infrared (FT-IR) spectroscopy

 FT-IR spectroscopy was used to characterize molecular interactions and structural changes occurring after sequential incorporation of GMS, CBD, 5-FU, CS, and HA. Figure 2 shows spectra for six formulations: (a) blank liposomes; (b) liposomes with 80 mg GMS; (c) liposomes with GMS and CBD; (d) liposomes with GMS, CBD, and 5-FU; (e) liposomes with GMS, CBD, 5-FU, and CS; and (f) liposomes with GMS, CBD, 5-FU, CS, and HA. Spectra, were recorded from 400 to 4000 cm^-1^ and key changes are indicated in the figure.

 The spectrum of blank liposomes (lecithin, cholesterol, and Tween 80) served as a reference.

 A broad band at 3200–3600 cm^-1^ was observed, attributed to O-H stretching

 (water or hydroxyl groups), while peaks at 2920–2850 cm^-1^ corresponded to C-H stretching of the alkyl chains in lecithin and cholesterol. A strong peak at 1740 cm^-1^ indicated C = O stretching of ester groups; peaks in the fingerprint region (1500–1000 cm^-1^) reflected characteristic phospholipid vibrations (Figure 2a).

 Adding 80 mg GMS increased intensity of the C-H stretching peaks (2920–2850 cm^-1^) and produced subtle changes in the C = O region (1740 cm^-1^), consistent with integration of GMS into the lipid matrix (Figure 2b). Encapsulation of CBD introduced additional hydroxyl and aromatic functionalities: The O–H band (3200–3600 cm^-1^) broadened and peaks in the 1600–1700 cm^-1^ region intensified, indicating CBD–lipid interactions (Figure 2c). Co-encapsulation of CBD and 5-FU produced further broadening of the O–H/N–H region and changes in the 1600–1700 cm^-1^ and fingerprint regions consistent with the presence of 5-FU (Figure 2d).

 Coating drug-loaded liposomes with CS introduced N–H and O–H bands and produced new or shifted features in the 1600–1650 cm^-1^ region attributable to amide groups, confirming CS deposition on the liposomal surface (Figure 2e). Final decoration with HA produced additional broadening of the O–H/N–H band and new/shifted peaks in the 1600–1700 cm^-1^ and fingerprint regions (1500–1000 cm^-1^), consistent with HA’s polysaccharide moieties and successful surface decoration (Figure 2f).

####  In vitro Release

 Both drugs exhibited faster release at pH 5.5 (endosomal-like conditions) than at pH 7.4 (physiological conditions).

**Figure 3:**
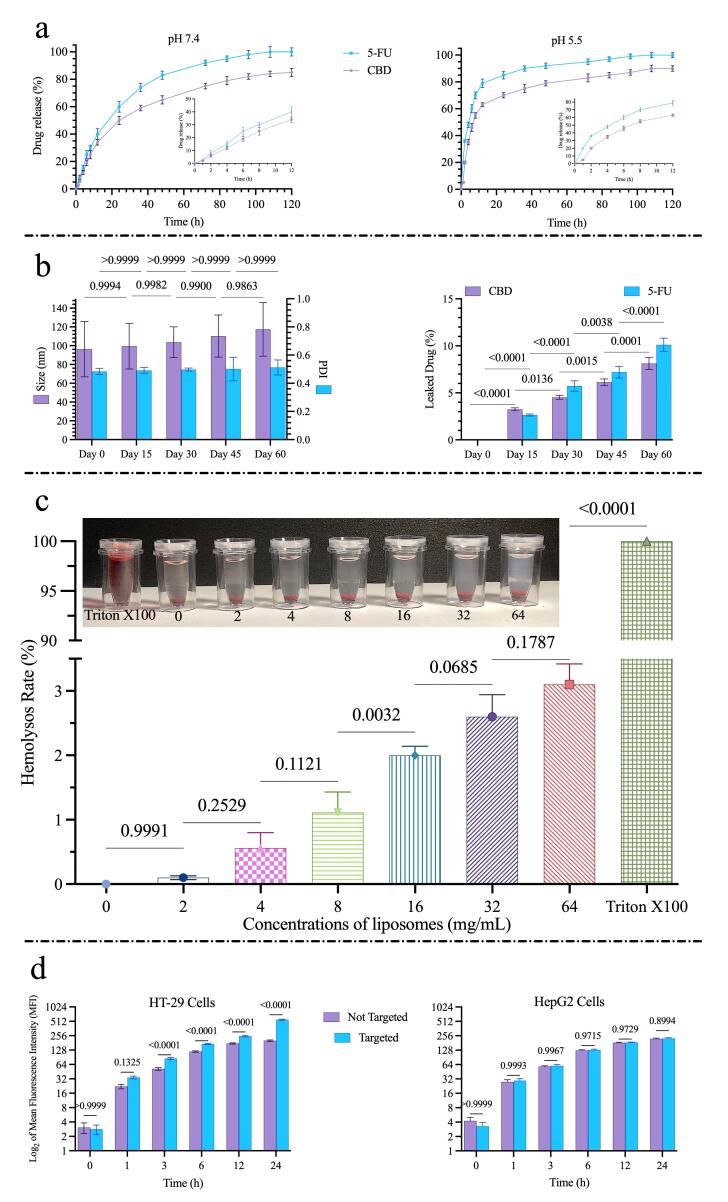
In vitro release, stability, and uptake of HA-functionalized liposomes. (a) Cumulative release profiles of 5-FU and CBD from formulations at pH 7.4 (physiological) and pH 5.5 (tumor-like acidic) over time. (b) Storage stability of the optimized formulation over 60 days at 4–8 °C: mean particle size, PDI, and drug leakage measurements at indicated time points. (c) Hemocompatibility (hemolysis) of HA-coated liposomes after incubation with human red blood cells at increasing concentrations. (d) Cellular uptake assessed by flow cytometry: mean fluorescence intensity (MFI) for liposomes labeled with fluorescein ± CS–HA decoration in HT-29 and HepG2 cells. Data are presented as mean ± SD (n = 3). Statistical comparisons are indicated on the panels

 At pH 5.5, cumulative release of 5-FU from HA-decorated liposomes reached ~79% after 12 h, whereas CBD release reached ~63% over the same period. At pH 7.4, cumulative release at 12 h was approximately 40% for 5-FU and 34% for CBD (Figure 3a). Release profiles for both drugs were biphasic, with an initial rapid phase followed by a slower sustained-release phase.

####  Release kinetics results

 The release kinetics of 5-FU and CBD from modified liposomes at pH 5.5 and pH 7.4 were assessed through multiple mathematical models, revealing insights into the underlying mechanisms. According to Table 6, the diffusion-relaxation model exhibited the highest correlation coefficients (R^2^ = 0.954–0.997), outperforming zero-order (R^2^ = 0.584–0.867), first-order (R^2^ = 0.39–0.58), second-degree polynomial (R^2^ = 0.759–0.973), Higuchi (R^2^ = 0.788–0.969), and Korsmeyer-Peppas (R^2^ = 0.890–0.976) models, which suggests a combined process of drug diffusion and liposomal matrix relaxation or erosion. The diffusion-relaxation model, often derived from extensions of the Korsmeyer-Peppas framework (such as the Peppas-Sahlin equation), accounts for two primary contributions to drug release: Fickian diffusion (where drug molecules move through pores or channels in the liposomal matrix driven by concentration gradients) and Case-II relaxation (involving swelling, or erosion of the matrix, which facilitates release by altering the structure over time).

**Table 6:** Results of kinetics models for 5 FU and CBD release

**Models**	**Parameters**	**CBD (pH 7.4)**	**5 FU (pH 7.4)**	**CBD (pH 5.5)**	**5 FU (pH 5.5)**
Zero order	K_0_	0.89	1.08	1.00	1.14
	R^2^	0.867	0.847	0.661	0.584
First order	K_1_	0.05	0.05	0.05	0.05
	R^2^	0.57	0.58	0.39	0.47
Second degree polynomial model	*K*_s1_	1.92 *K*_s1_	2.41	2.62	3.18
	*K*_s2_	-0.01	-0.01	-0.02	-0.02
	R^2^	0.971	0.973	0.814	0.759
Higuchi	*K*_H_	8.59	10.21	6.94	6.47
	C	-0.58	0.62	23.51	39.65
	R^2^	0.969	0.958	0.825	0.788
Korsmeyer-Peppas	*K*_kp_	0.10	0.11	0.28	0.38
	n	0.502	0.493	0.289	0.226
	R^2^	0.976	0.972	0.890	0.890
Diffusion relaxation	*K*_d_	0.07	0.07	0.22	0.32
	*K*_d_	-1.15 × 10^-3^	-1.12 × 10^-3^	-0.01	-0.03
	n	0.733	0.755	0.512	0.435
	R^2^	0.994	0.997	0.954	0.966

 In this model, the parameters typically include a diffusion constant (e.g., 0.32 for 5-FU at pH 5.5, indicating the rate of diffusional release), a relaxation constant (e.g., -0.03, where negative values might suggest minimal or opposing relaxation effects in certain conditions), and an exponent n (e.g., 0.435 at pH 5.5 for 5-FU, reflecting the relative balance between diffusion and relaxation mechanisms—values closer to 0.5 indicate diffusion dominance, while higher values point to increased relaxation involvement). At pH 7.4, R^2^ values were consistently higher for most models compared to pH 5.5, indicating more predictable and sustained release under neutral conditions, while the acidic environment appeared to accelerate initial release rates.

###  Formulation stability during storage

 The stability of the final formulations was assessed over 60 days of storage by monitoring changes in particle size, PDI, and drug leakage. As shown in Figure 3b, the mean particle size increased slightly from 96.35 nm to 117.0 nm during the storage period; however, this change was not statistically significant (*P* = 0.5893). Similarly, the PDI value showed a minor variation from 0.484 to 0.512, which was also not significant (*P* > 0.9999), indicating that the formulations maintained good homogeneity. In contrast, the drug leakage study revealed low levels of drug loss for both agents. After 60 days, the cumulative leakage was approximately 8.12% for CBD and 10.11% for 5-FU. Overall, these findings demonstrate that the formulations exhibited satisfactory physical stability and drug retention during refrigerated storage.

####  Hemolysis assay

 Hemolysis results are presented in Figure 3c. The percentage of hemoglobin released from RBCs after exposure to nanoformulation at concentrations of 2, 4, 8, 32, 64 mg/mL was measured. No significant hemolysis was observed at the tested concentrations; the highest concentration (64 mg/mL) induced only 3.1% hemolysis, which is negligible relative to the positive control (1% Triton X100), defined as 100% hemolysis.

###  Cell uptake

 Uptake of plain and HA-targeted liposomes was compared in HT-29 and HepG2 (Figure 3d). HT-29 cells (high CD44 expression) and HepG2 cells (lower CD44 expression) were incubated with fluorescein-labeled formulations for 1, 3, 6, 12, and 24 h.

 Uptake by HT-29 cells was time dependent, and HA-targeted liposomes demonstrated significantly greater uptake than plain liposomes (*P* < 0.0001). in HepG2 cells, both targeted and non-targeted liposomes were internalized, but no significant difference in uptake was observed (*P* > 0.05). These data indicate that HA decoration enhance cellular internalization via CD44-mediated endocytosis in CD44-overexpressing cells.

###  Cellular cytotoxicity studies

**Figure 4:**
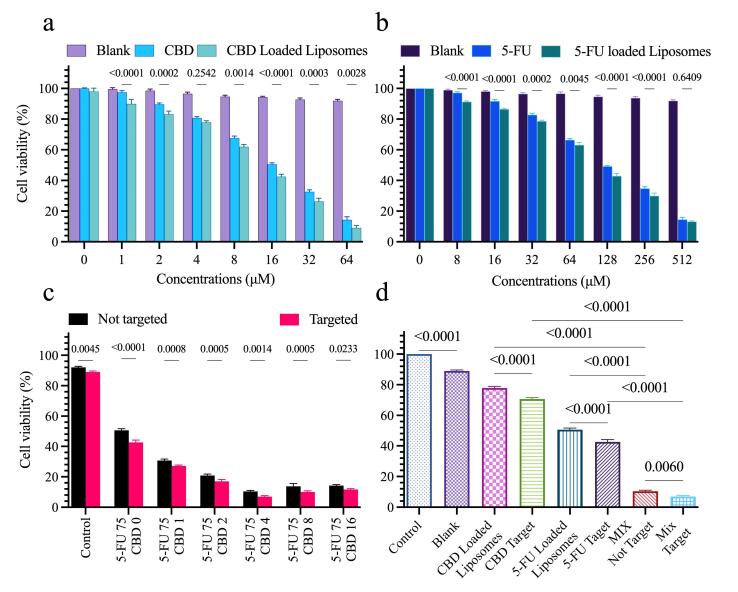
Cytotoxicity of formulations in HT-29 cells. Dose–response curves showing percent cell viability after treatment with: (a) cannabidiol (CBD) formulations, (b) 5-fluorouracil (5-FU) formulations, (c) co-formulated 5-FU + CBD in non-targeted and HA-targeted liposomes across tested molar ratios, and (d) summary comparison of viability for all formulations at the selected concentration(s). Data are mean ± SD (n = 3). IC₅₀ values (where calculated) are reported in the main text or figure inset

 Cytotoxicity of blank liposomes, free CBD, CBD-loaded liposomes, free 5-FU, 5-FU-loaded liposomes, and combined formulations (targeted and non-targeted) was evaluated in HT-29 cells using the MTT (Figure 4).

 This study proceeded in two stages. In the first stage, the effect of liposomal encapsulation on single-agent potency was assessed. Blank liposomes exerted minimal (cell viability > 80%), indicating good biocompatibility (Figures 4a, 4b). Encapsulation of CBD reduced its IC_50 _from 16 μM to 8 μM, indicating enhanced potency likely due to controlled release and improved cellular delivery. Similarly, encapsulation of 5-FU lowered its IC_50 _from 117 μM to 75 μM (Figure 4a), suggesting improved intracellular availability and pH-responsive release.

 In the second stage, the IC_50_ of 5-FU-loaded liposomes (75 μM) was fixed as a constant concentration to assess synergistic effects of increasing CBD concentrations. Keeping 5-FU constant at 75 μM, cells were treated with liposomal formulations at 5-FU:CBD molar ratios of 75:1, 75:2, 75:4, 75:8, and 75:16 (μM: μM). This design allowed systematic evaluation of how incremental CBD concentrations modulate cytotoxicity. Combination treatment markedly reduced cell viability, with the 75:4 (5-FU:CBD) ratio producing the greatest decrease in viability. Higher CBD ratios (75:8, 75:16) also reduced viability but the 75:4 ratio was identified as optimal. Furthermore, HA-mediated active targeting increased cytotoxic efficacy by an average of ~26%, underscoring the contribution of receptor-mediated uptake to therapeutic performance (Figure 4d).

###  ROS production and colony formation

**Figure 5:**
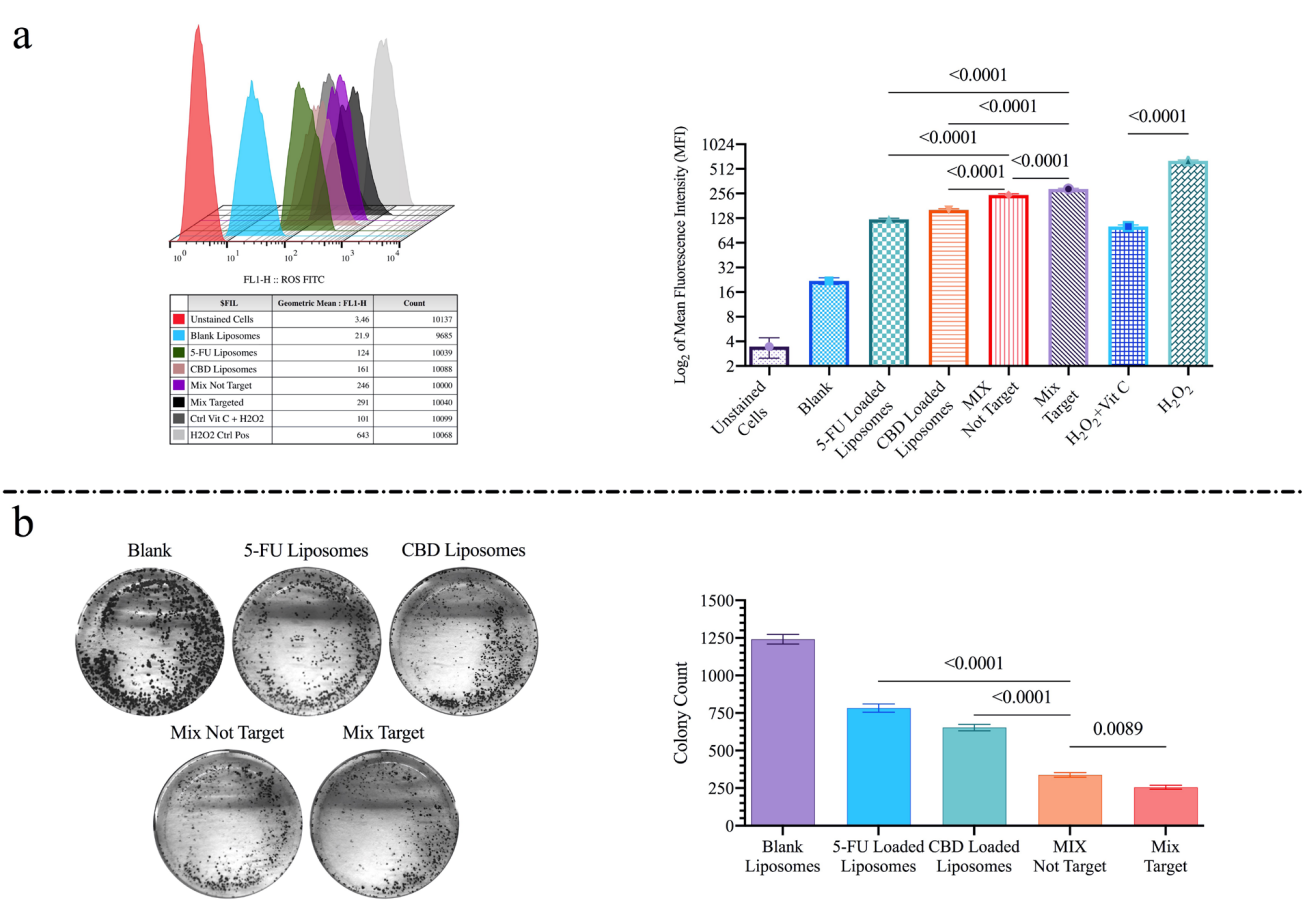
Oxidative stress induction and long-term proliferative potential. (a) Flow cytometry analysis of intracellular reactive oxygen species (ROS) in HT-29 cells following treatment with indicated formulations (data expressed as mean fluorescence intensity or percent ROS-positive cells). (b) Representative images and quantification of colony formation assay showing the antiproliferative effect of each formulation after colony development. Data are presented as mean ± SD (n = 3). Representative wells/images are shown; quantitative results are plotted

 Intracellular ROS generation was measured using DCFH-DA and flow cytometry. Encapsulating 5-FU and CBD together in liposomes increased ROS production by 73% relative to liposomes loaded with either agent alone (*P* < 0.0001; Figure 5a). HA targeting the mixed formulation further enhanced ROS production by ~18% compared with non-targeted (*P* < 0.0001).

 Colony-formation assay assessed long-term proliferative capacity. The combined 5-FU/CBD liposomes produced a substantially greater reduction in colony formation than single-agent liposomes; specifically, the combination resulted in a ~52% reduction in colony formation relative to liposomes loaded with either 5-FU or CBD alone (*P*< 0.0001). HA-targeted liposomes reduced colony formation by an additional ~24% compared with non-targeted mixed liposomes (*P* = 0.0089; Figure 5b).

###  Apoptosis (Annexin V/PI) and DAPI staining 

**Figure 6:**
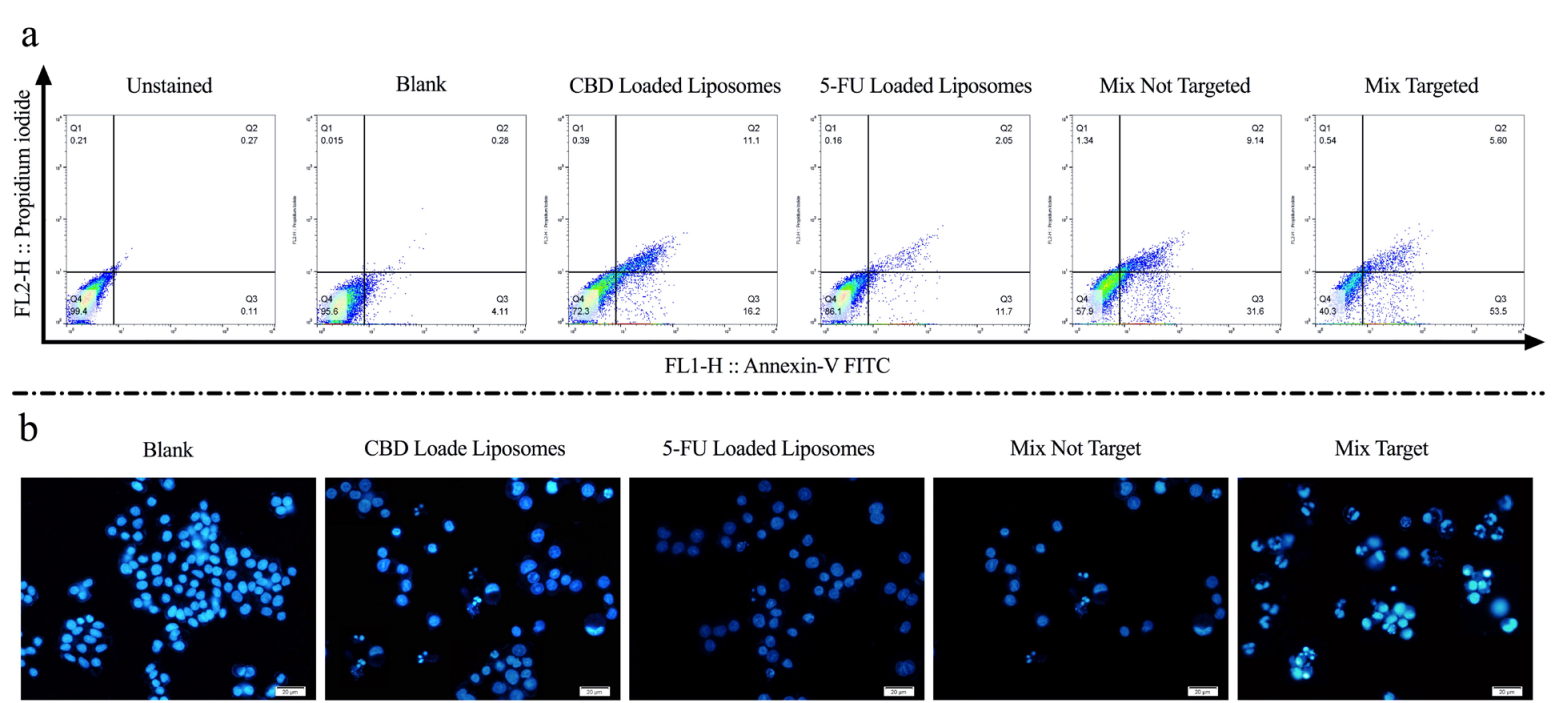
Induction of apoptosis and nuclear morphology. (a) Annexin V/PI flow cytometry quantification of viable, early apoptotic, late apoptotic, and necrotic HT-29 cell populations after treatment with different formulations. (b) Representative DAPI-stained micrographs of HT-29 cells post-treatment ( × 400 magnification); nuclear condensation and fragmentation are indicated. Quantitative apoptosis data are presented as mean ± SD (n = 3). Scale bars are shown in the images

 Apoptosis and necrosis were quantified by Annexin V/PI staining (Figure 6a). CBD-loaded liposomes induced total apoptosis of 27.3%, whereas 5-FU-loaded liposomes induced 13.75% apoptosis. Co-encapsulation of 5-FU and CBD increased apoptosis to 40.74%, and HA targeting of the mixed formulation further raised the apoptosis rate to 59.1% (*P* < 0.0001). Blank liposomes induced minimal apoptosis (4.39%).

 DAPI staining (Figure 6b) revealed chromatin condensation and nuclear fragmentation consistent with apoptosis. Both single-agent formulations caused nuclear fragmentation, which was more pronounced in the comminuted formulation; the most extensive fragmentation was observed with HA-targeted mixed-drug liposomes.

###  Cell cycle analysis

**Figure 7:**
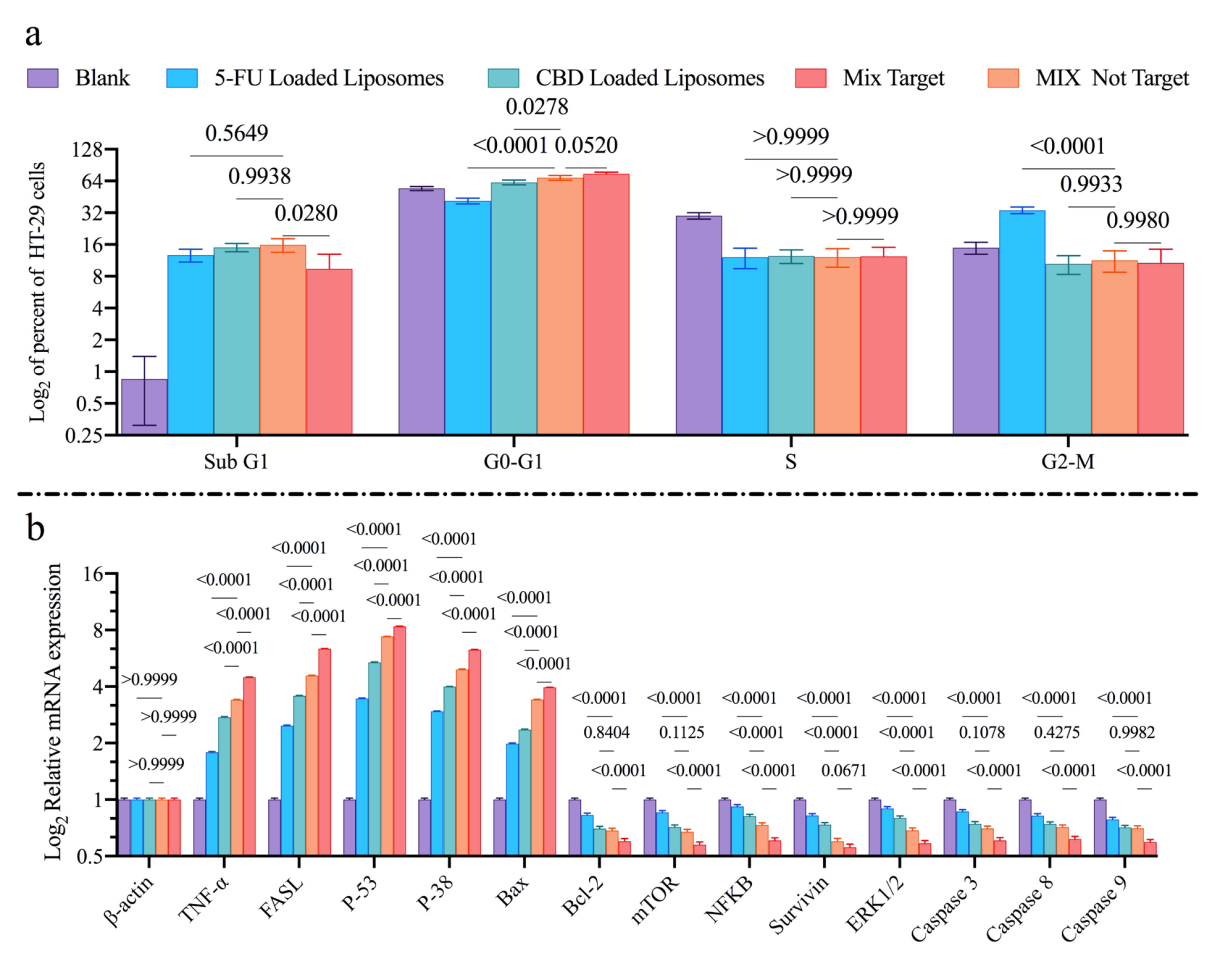
Cell-cycle distribution and transcriptional responses. (a) Cell-cycle phase distribution (G0/G1, S, G2/M) of HT-29 cells after treatment with indicated formulations (flow cytometry). (b) Relative mRNA expression levels of genes associated with apoptosis and proliferation (e.g., p53, BAX, BCL-2, TNF-α, mTOR, NF-κB, ERK1/2) measured by qRT-PCR and normalized to housekeeping gene(s). Data are presented as mean ± SD (n = 3); fold changes and statistical comparisons are indicated in the panels or figure legend

 Cell cycle distribution was analyzed after 48 h treatment (Figure 7a). CBD-loaded liposomes produced only a small increase in the SubG1 population compared with blank liposomes. In contrast, 5-FU-loaded liposomes induced marked G2-M arrest (33.8% of cells).

 Mixed-drug liposomes, produced the most pronounced G0–G1 arrest (68.63% of cells), exceeding the arrest induced by either single agent. Active targeting with HA further increased G0–G1 arrest from 68.63% to 74.58%. The proportion of cells in S phase remained largely unchanged across treatments.

###  Gene expression (qRT -PCR) evaluation

 Quantitative real-time PCR was used to assess expression of apoptosis- and cell cycle-related genes (Figure 7b). Treatment with CBD- or 5-FU-loaded liposomes upregulated mRNA levels of TNF-α, FASL, P-53, P-38, and Bax, while downregulating expression of Bcl-2, mTOR, NFKB, Survivin, ERK1/2, Caspase3, Caspase8, and Caspase9. HA-targeted mixed-drug liposomes were produced more pronounced modulation of these genes than non-targeted formulations. Cells treated with blank liposomes showed no significant changes in the expression of the evaluated genes.

## Discussion

 CRC remains the third most prevalent cancer worldwide and continues to present significant therapeutic challenges because of its high incidence and mortality.^27^ Current treatment regimens typically combine surgery with systemic chemotherapy; however, chemotherapy efficacy is often limited by off-target toxicity and emergence of tumor resistance.^23^ Combination chemotherapy is therefore widely pursued to enhance antitumor efficacy while reducing adverse side effects.^28^ In this context, nanoparticle-based delivery systems particularly ligand-decorated liposomes offer a route for selective drug delivery to cancer cells and minimize systemic toxicity.^26^ 5-FU is a cornerstone of CRC chemotherapy but is constrained by dose-dependent toxicity and acquired resistance.^29^ To overcome these limitations, several groups have explored HA-modified liposomal carriers for 5-FU delivery and reported improved cellular targeting and gene modulation, although many studies lack comprehensive *in vivo* validation.^2^. Concurrently, CBD has attracted interest for its anticancer properties; nanoformulated CBD improves its stability and antitumor activity in preclinical models, and other studies implicate CBD in the modulation of signaling pathways that suppress CRC progression.^30,31^

 In present study, we developed HA-decorated, chitosan-modified liposomes for co-delivery of 5-FU and CBD, exploiting HA’s affinity for CD44 receptors that are overexpressed on many CRC cells.^27,32^ Our formulations exhibited a narrow size distribution (mean diameter ≈ 96 nm; PDI < 0.3), a size range compatible with efficient tumor penetration and cellular internalization (Figure 1).^33^ Surface modification with HA shifted the zeta potential from + 40.10 mV to −1.53 mV (Figure 1), consistent with successful HA decoration and with a reduction in net surface charge that is expected to improve biocompatibility.^27^ Although negatively charged surface typically reduce nonspecific adhesion, interactions with cationic domains on the cell membrane can promote nanoparticle clustering and augment cellular uptake, which may explain the effective internalization in our system.^34,35^

 Drug release studies demonstrated pH-dependent kinetics (Figure 3b): both 5-FU and CBD were released more rapidly at pH 5.5 than at pH 7.4 consistent with a design intended to favor intracellular release and minimize premature systemic exposure.^36^ The release kinetics and stability data together indicate a favorable profile for the HA-functionalized liposomes as a co-delivery vehicle. Quantitative fitting showed the diffusion–relaxation model (R^2^ = 0.954–0.997) provided the best description of both 5-FU and CBD release, implying that drug liberation is governed by a combination of Fickian diffusion through the lipid matrix and structural relaxation/erosion of the carrier; the calculated diffusion constants (for example, ~0.32 for 5-FU at pH 5.5) and n values near 0.43–0.5 point to a predominance of diffusion with a measurable contribution from relaxation.^37^ Release was more predictable and sustained at pH 7.4, whereas the acidic condition (pH 5.5) accelerated early release, a behavior that is advantageous for preferential intracellular/endosomal drug delivery in the tumor microenvironment.^38^ Importantly, these functional release characteristics were achieved without compromising short-term physical stability: particle size and PDI remained essentially unchanged over 60 days of refrigerated storage, and cumulative drug leakage remained low (≈8.1% for CBD and ≈10.1% for 5-FU). Collectively, these findings suggest the formulation can retain payload during storage yet release drugs responsively under tumor-like acidic conditions, supporting its potential utility for targeted intracellular delivery, while recognizing that in vivo validation will be required to confirm these advantages. The formulation also achieved high encapsulation efficiencies and relatively small particle size compared with similar systems reported in the literature.^27^ Hemocompatibility testing indicated that our system has low hemolysis ( <4% at 64 mg/mL), supporting that, this formulation is biocompatible for *in vivo* applications.^39^

 Cellular uptake studies confirmed CD44-mediated internalization in HT-29 but not HepG2 cells (Figure 3c), supporting the active ligand-based targeting capability of our formulations.^40^ This receptor-mediated uptake likely contributes to the enhanced intracellular delivery of both drugs and helps explain downstream functional effects.

*In vitro* cytotoxicity assays showed that HA-targeted, dual-drug liposomes produced significantly greater reductions in HT-29 cell viability than single-agent or non-targeted formulations (Figure 4). Blank liposomes exhibited minimal toxicity ( > 80% viability), confirming the biocompatible nature of the carrier.^17^ Annexin V/PI assay and DAPI staining (Figure 6a,b) corroborated these findings: co-encapsulation of 5-FU and CBD increased apoptotic indices relative to single agents, and HA targeting further augmented apoptosis (maximum observed apoptosis ≈59.1%). Together these results indicate that targeted co-delivery improves both the degree and the specificity of cytotoxicity in CD44-expressing CRC cells.

 Cell cycle analysis revealed complementary effects of two drugs (Figure 7a). Liposomal 5-FU induced prominent G2/M arrest (33.8%), whereas CBD liposomes induced a G0/G1 arrest (62.13%). The not-targeted mixed-drug formulation produced G0/G1 arrest (68.62%), suggesting that the drugs act through partially distinct antiproliferative mechanisms that converge to inhibit proliferation. Targeting the formulation with HA increased G0/G1 arrest by 5.96 % relative to the non-targeted form, which may result from enhanced cellular internalization of targeted liposomes and thereby greater intracellular drug delivery.^20^

 Colony formation assays and ROS measurements supported a synergistic cytotoxic effect for the combination with HA targeting, demonstrating enhanced long-term growth suppression and increased ROS generation (Figure 5a,b). Mechanistically, CBD and 5-FU appear to act through complementary pathways that amplify oxidative stress and impair clonogenic survival. CBD can induce ROS production by disrupting mitochondrial function and promoting endoplasmic reticulum stress, which elevates intracellular ROS and triggers autophagy and apoptosis ^41^. Likewise, 5-FU contributes to ROS generation via incorporation into RNA and DNA, causing replication stress and secondary mitochondrial dysfunction.^42^ When combined, these agents increase ROS accumulation beyond the compensatory capacity of antioxidant systems (e.g., glutathione peroxidase), resulting in oxidative damage to lipids, proteins, and DNA.^43^ This amplified ROS burden activates downstream effectors—including ERK phosphorylation and caspase-dependent apoptosis—leading to cell cycle arrest and programmed cell death.^41^ Consequently, the combination markedly inhibits colony formation, a readout of long-term clonogenic potential, and mirrors findings in other tumor models where CBD lowers the effective concentration of standard chemotherapeutics required for cytotoxicity.^44^ Overall, these data support CBD as an adjuvant that enhances 5-FU efficacy by exploiting ROS-mediated vulnerabilities in cancer cells.

 qRT-PCR analysis further supported induction of apoptotic programs and suppression of survival signaling (Figure 7b): combined treatment upregulated pro-apoptotic genes (TNF-α, FASL, p53, BAX) and downregulated survival genes (BCL-2, mTOR, NF-κB, ERK1/2). This transcriptional profile is consistent with activation of both intrinsic and extrinsic apoptotic pathways TNF-α/FASL engaging death receptor signaling while p53/BAX promote mitochondrial permeabilization and caspase activation. Suppression of BCL-2 disrupts anti-apoptotic buffering, and inhibition of mTOR, NF-κB, and ERK1/2 curbs proliferative and survival cascades together providing a molecular rationale for the enhanced cytotoxicity of the combined, targeted formulation and aligning with prior observations in HNSCC models.^45-47^

 Several limitations should be acknowledged. First, these results are restricted to *in vitro* assays and therefore do not capture the full complexity of an *in vivo* environment (e.g., hemodynamics, protein corona formation, immune interactions, and biodistribution). Second, the differing release kinetics of 5-FU and CBD present challenges for co-administration that will require optimization of dosing schedules and possibly further formulation refinements to synchronize effective intracellular exposure. Third, the observed changes in caspase expression and other apoptotic markers may be time-dependent; more comprehensive temporal profiling and protein-level analyses (e.g., Western blot, caspase activity assays) are necessary to define precisely which pathways are engaged and when. Finally, long-term toxicity, immunogenicity, and potential resistance mechanisms were not assessed here and will be essential for translational evaluation.

## Conclusion

 This study establishes HA-decorated liposomes as a potential platform for the co-delivery of 5-FU and CBD CRC-targeted applications. By synergistically enhancing apoptosis, cell cycle arrest, and oxidative stress while minimizing off-target effects, this system represents a translatable strategy for precision oncology. Further preclinical validation is warranted to advance this therapeutic paradigm.

## Competing Interests

 The authors of the manuscript entitled “Targeted Co-delivery of 5-Fluorouracil and Cannabidiol via Chitosan-Modified Hyaluronic Acid-Decorated Liposomes for an Augment Colorectal Anticancer Effects” declare no conflicts of interest about the submitted manuscript. This declaration of interest is made to the best of our knowledge and belief, and we understand that any significant undisclosed conflicts of interest may lead to the withdrawal of the manuscript from consideration for publication.

## Data Availability Statement

 All data that support our findings in this study are available from the corresponding authors upon reasonable demand.

## Ethical Approval

 The research protocol was approved by the Tabriz University of Medical Sciences Biomedical Research Ethics Committee (approval no. IR.TBZMED.VCR.REC.1404.046). Human blood samples were collected from consenting volunteers and processed according to institutional procedures. All methods were performed in accordance with relevant guidelines and regulations, including the Declaration of Helsinki. Cell lines were handled following institutional biosafety policies.
